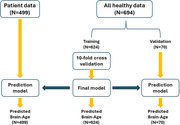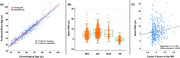# Neuropsychiatric symptoms and brain aging in mild cognitive impairment and early dementia: A multicenter study

**DOI:** 10.1002/alz.093747

**Published:** 2025-01-09

**Authors:** Daichi Sone, Iman Beheshti, Kenji Tagai, Hiroshi Kameyama, Emi Takasaki, Tetsuo Kashibayashi, Ryuichi Takahashi, Kazunari Ishii, Hideki Kanemoto, Manabu Ikeda, Masahiro Shigeta, Shunichiro Shinagawa, Hiroaki Kazui

**Affiliations:** ^1^ Jikei University School of Medicine, Minato‐ku, Tokyo Japan; ^2^ University of Manitoba, Winnipeg, MB Canada; ^3^ Hyogo Prefectural Rehabilitation Hospital at Nishi‐Harima, Tatsuno, Hyogo Japan; ^4^ Kindai University Faculty of Medicine, Osakasayama, Osaka Japan; ^5^ Osaka University Graduate School of Medicine, Suita, Osaka Japan; ^6^ Kochi University, Nankoku, Kochi Japan

## Abstract

**Background:**

Despite the clinical importance and significant social burden of neuropsychiatric symptoms (NPS) in dementia, the underlying neurobiological mechanism remains poorly understood. Recently, neuroimaging‐derived brain‐age estimation by machine‐learning analysis has shown promise as an individual‐level biomarker. We investigated the relationship between NPS and brain‐age in amnestic mild cognitive impairment (MCI) and early dementia.

**Method:**

Clinical data, including neuropsychiatric inventory (NPI), and structural brain MRI of 499 individuals with clinical diagnoses of amnestic MCI (N=185), early Alzheimer’s disease (AD) (N=258) or dementia with Lewy bodies (DLB) (N=56) were analyzed. We established a brain‐age prediction model using 694 brain structural MRI scans of healthy subjects and support vector regression model and applied it to the patients’ data. Finally, the brain predicted age difference (brain‐PAD: predicted age minus chronological age) were calculated.

**Result:**

All clinical diagnostic groups showed significantly increased brain‐PAD, and the median (IQR) brain‐PAD was 4.3 (5.4) years in MCI, 6.3 (6.2) years in AD, and 5.0 (6.5) years in DLB. The NPI scores were subdivided into the following four categories: (i) Agitation and Irritability, (ii) Depression and Apathy, (iii) Delusions and Hallucinations, and (iv) Euphoria and Disinhibition. We found a significantly positive correlation between brain‐PAD and the depression/apathy factor (Spearman’s rs = 0.156, FDR‐corrected p=0.002), while no significance was shown in the other NPS factors.

**Conclusion:**

Abnormal brain aging may be involved in depression and apathy symptoms presented in MCI to early dementia stages, and brain‐age analysis may be useful as a novel biomarker for assessment or monitoring of NPS.